# Characterization and mutational analysis of a nicotinamide mononucleotide deamidase from *Agrobacterium tumefaciens* showing high thermal stability and catalytic efficiency

**DOI:** 10.1371/journal.pone.0174759

**Published:** 2017-04-07

**Authors:** Ana Belén Martínez-Moñino, Rubén Zapata-Pérez, Antonio Ginés García-Saura, Fernando Gil-Ortiz, Manuela Pérez-Gilabert, Álvaro Sánchez-Ferrer

**Affiliations:** 1 Department of Biochemistry and Molecular Biology-A, Faculty of Biology, Regional Campus of International Excellence “Campus Mare Nostrum”, University of Murcia, Campus Espinardo, E-30100 MURCIA, Spain; 2 Murcia Biomedical Research Institute (IMIB), Murcia, Spain; 3 CELLS-ALBA Synchrotron Light Source, Barcelona, Spain; National Renewable Energy Laboratory, UNITED STATES

## Abstract

NAD^+^ has emerged as a crucial element in both bioenergetic and signaling pathways since it acts as a key regulator of cellular and organismal homeostasis. Among the enzymes involved in its recycling, nicotinamide mononucleotide (NMN) deamidase is one of the key players in the bacterial pyridine nucleotide cycle, where it catalyzes the conversion of NMN into nicotinic acid mononucleotide (NaMN), which is later converted to NAD^+^ in the Preiss-Handler pathway. The biochemical characteristics of bacterial NMN deamidases have been poorly studied, although they have been investigated in some firmicutes, gamma-proteobacteria and actinobacteria. In this study, we present the first characterization of an NMN deamidase from an alphaproteobacterium, *Agrobacterium tumefaciens* (AtCinA). The enzyme was active over a broad pH range, with an optimum at pH 7.5. Moreover, the enzyme was quite stable at neutral pH, maintaining 55% of its activity after 14 days. Surprisingly, AtCinA showed the highest optimal (80°C) and melting (85°C) temperatures described for an NMN deamidase. The above described characteristics, together with its high catalytic efficiency, make AtCinA a promising biocatalyst for the production of pure NaMN. In addition, six mutants (C32A, S48A, Y58F, Y58A, T105A and R145A) were designed to study their involvement in substrate binding, and two (S31A and K63A) to determine their contribution to the catalysis. However, only four mutants (C32A, S48A Y58F and T105A) showed activity, although with reduced catalytic efficiency. These results, combined with a thermal and structural analysis, reinforce the Ser/Lys catalytic dyad mechanism as the most plausible among those proposed.

## Introduction

**Fig 1:**
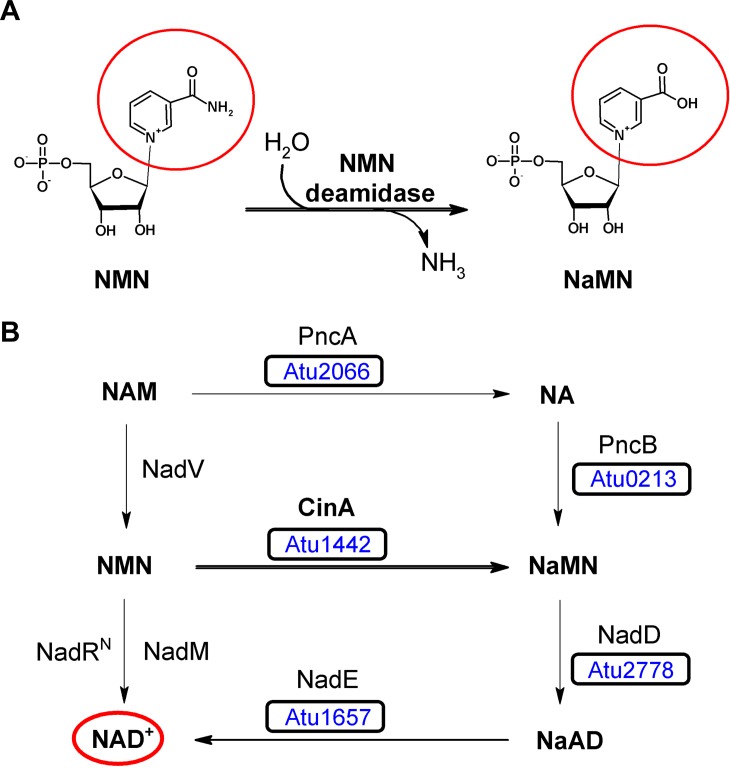
NMN deamidase activity and NAD^+^ salvage pathway in *A*. *tumefaciens*. A) Nicotinamide mononucleotide deamidase reaction. NMN is enzymatically transformed into nicotinic acid mononucleotide and ammonia. B) NAD^+^ salvage pathway in *A*. *tumefaciens*. Enzymes are indicated by the acronym used to identify the corresponding gene name. NadD, NaMN adenylyltransferase; NadE, NAD synthetase; NadM, NMN adenyltransferase; NadR^N^, NMN adenylyltransferase; NadV, NAM phosphoribosyltransferase; PncA, nicotinamidase; PncB, nicotinic acid phosphoribosyltransferase; CinA, nicotinamide mononucleotide deamidase. The NCBI locus tag for each enzyme in *A*. *tumefaciens* is shown inside the corresponding square. NAD^+^, nicotinamide adenine dinucleotide; NAM, nicotinamide; NA: nicotinic acid; NaMN, nicotinic acid mononucleotide; NMN, nicotinamide mononucleotide; and NaAD, nicotinic acid adenine dinucleotide.

During the last decade, it has become clear that the cellular role of NAD^+^ extends far beyond its classic participation in redox reactions, since it also acts as a substrate of several families of regulatory enzymes [1–4]. For this reason, intracellular concentrations of many NAD^+^ intermediates are maintained low [5–8], and NAD^+^ is considered as a sensor of metabolic and cellular stress [9–11]. Regardless of the origin of these NAD^+^-consuming enzymes, many other enzymes are involved in preserving the NAD^+^ supply, ranging from those related with its *de novo* synthesis to those associated with its regeneration in accordance with the pyridine nucleotide cycle [12–15]. Among them, the enzyme nicotinamide mononucleotide (NMN) deamidase or amidohydrolase (EC 3.5.1.42) was an ´orphan enzyme´ [16] with no associated sequence for many years. It was first mentioned in 1969, as an enzyme in *Clostridium sticklandii* responsible of deaminating NMN to nicotinic acid mononucleotide (NaMN) (**Fig 1A**) [17]. Finally, its gene (*pncC*) was sequenced in the marine bacterium *Shewanella oneidensis* MR-1 [18], and matched with a family of genes annotated in databases as competence/damage-inducible protein A (*cinA*). This name derives from the fact that in some microorganisms these *cinA* genes are found adjacent to the competence gene *recA*, whose product is one of the proteins described in the DNA uptake and incorporation (´competence´) pathway. The function of CinA protein in this process is still a riddle, although it could play a dual role in decreasing the intracellular level of NMN to avoid inhibition of NAD^+^-dependent DNA ligases, and at the same time, securing a continuous NAD^+^ supply for the ligases by propelling NaMN into the Preiss-Handler pathway to produce new NAD^+^ [18].

Few data exist in the bibliography about these NMN deamidases. In fact, only five have been kinetically characterized [13,18–21] and their nomenclature is confusing, since two of the terms “PncCs” and “CinAs” are used indistinctively, while they are in fact different. Phylogenetic and domain analyses have shown that the term PncC is better applied to enzymes formed by a domain with NMN deamidase activity (CinA domain) fused to another domain with ADP-ribose (ADPr) pyrophosphatase or NUDIX (Nucleoside Diphosphate linked to X) hydrolase activity (MocF domain), whereas the term CinA is better applied to NMN deamidases with only one domain [18,19].

Currently, only one structure of each kind of enzyme was determined, a CinA from the bacterium *Agrobacterium tumefaciens* (PDB code: 2A9S), and a PncC from *Thermus thermophilus* (TtPncC) (PDB codes: 4UOC, 4CT8, 4UUX, 4CTA and 4UUW) [22]. In addition, a MocF domain structure has also been resolved from *Thermoplasma acidophilum* (PDB code: 3KBQ). ADPr and NMN substrates were docked to the above structures [18,19], and NaMN and ADPr were co-crystallized [22] in order to identity the amino acids involved in binding and catalysis. Based on these latter studies and the mutational analysis carried out with *E*. *coli* CinA [23], two different catalytic mechanisms have been proposed [22,23], one describing a catalytic Ser/Lys dyad mechanism [23] and the other suggesting an asparaginase II-like mechanism [24]. In this last mechanism, a water nucleophilic attack is promoted by a lysine (K301 in TtPncC) on the amide bond, and the resulting tetrahedral intermediate is rearranged to eliminate ammonia with the assistance of a threonine (T340 in TtPncC) [22].

In order to expand our limited knowledge of NMN deamidases, especially in those with only one domain, a detailed biochemical and mutational study was carried out with the NMN deamidase from *Agrobacterium tumefaciens* (AtCinA). This enzyme was selected because it belongs to a biochemically uncharacterized subgroup (2.2.3) in the general phylogenetic tree described for the NMN deamidase family [19]. In addition, no NMN deamidases from an alphaproteobacteria have been previously characterized. And finally, its activity could play an important role in the NAD^+^ homeostasis in *Agrobacterium tumefaciens*, since no NMN adenyltransferase (NadM or NadR) is present in this microorganism to recycle NMN into NAD^+^ (**Fig 1B**). The results obtained showed that AtCinA not only has high stability at neutral pHs, but also the highest optimal temperature (80°C) described in the bibliography for NMN deamidases. This high stability, corroborated by its high melting temperature (85°C), and its high catalytic efficiency, make AtCinA a promising biocatalyst for obtaining pure NaNM from NMN, in a process of potential commercial interest, given that the commercial value of the product is 10 times that of the substrate. In addition, the correlation observed between the results of the mutational analysis and the enzyme structure sheds light on the NMN deamidase catalytic mechanism.

## Materials and methods

### Strains, plasmids, and chemicals

*cinA* gene from *Agrobacterium tumefaciens* was obtained from GenScript (Piscataway, USA). The pET24b cloning vector was from Novagen (EMD Millipore, Madrid, Spain). QIAquick PCR purification kit and QIAprep spin miniprep kit were from Qiagen (Valencia, CA, USA). KAPA HiFi polymerase was from KapaBiosystems (Boston, MA, USA). NAD^+^ was from Carbosynth (Berkshire, UK). Nicotinamide mononucleotide and nicotinic acid mononucleotide were from Santa Cruz Biotechnology (Heidelberg, Germany). Other reagents were obtained from Sigma-Aldrich (Madrid, Spain).

### Cloning of the AtCinA gene and mutants

The cloning and transformation techniques used were essentially those previously described [25]. The 510 bp synthetic gene from *A*. *tumefaciens* was amplified by PCR using KAPA HiFi DNA polymerase and the corresponding primers (**S1 Table**), including *Nhe*I and *Xho*I restriction site extensions. The resulting PCR product was purified and digested with the above mentioned restriction enzymes, ligated to the digested pET24b (C-terminal His tag), and transformed into *Escherichia coli* Rosetta™ 2(DE3) competent cells. A specific clone harboring the correct sequence was denoted as pET24b-AtCinA.

Site-directed mutagenesis of AtCinA was carried out using the QuikChange XL Site-Directed Mutagenesis Kit (Agilent Technologies), according to the manufacturer’s instructions. Sequences of the mutagenic primers are listed in **S1 Table**. The mutagenized plasmids were sequenced to verify that the desired modification had been incorporated and to ensure the absence of random mutations.

### Expression and purification of AtCinA and its mutants

The above *E*. *coli* cells harboring the recombinant plasmid pET24b-AtCinA and its corresponding mutants were grown for 3 hours at 37°C in 100 mL Luria Broth (LB) containing 50 μg/mL kanamycin and 34 μg/mL chloramphenicol before being transferred to a 2.5 L culture flask containing 1 L of LB supplemented with the above antibiotics. These cultures were grown for 3h at 37°C until an OD_600_ of 0.8 was reached, and then induced with 0.5 mM isopropyl-β-D-thiogalactoside (IPTG) for 6 hours at 37°C with constant shaking. Pelleted cells were resuspended in 50 mM Tris-HCl pH 8.0, 200 mM NaCl, 10% (v/v) B-PER (Bacterial Protein Extraction Reagent, Thermofisher), 1 mg/mL lysozyme and 1 mM phenylmethylsulfonyl fluoride (PMSF, Sigma) before being disrupted by sonication (450-D Sonifier, Branson). After ultracentrifugation (40000*g*, 40 min), the resulting supernatant was purified by anion ion exchange (HiPrep Q column) followed by Ni^2+^-chelating affinity chromatography (HiPrep IMAC 16/10 FF column) (GE Life Sciences). The fractions containing the NMN deamidase activity were pooled, desalted, concentrated and stored at -80°C with 10% glycerol. Gel filtration (Superdex 200 10/300 GL, GE Life Sciences) was used to confirm the homogeneity and the molecular mass of the purified enzyme. The protein concentration was determined using Bradford´s reagent (Bio-Rad) and BSA as standard.

### Enzyme assay

AtCinA activity was determined by an HPLC-based assay, which follows the decrease in area of the NMN peak, using a C_18_ column (Phenomenex Gemini C_18_, 4.6 x 250 mm) and a mobile phase (20 mM ammonium acetate pH 6.9) running at 0.8 mL/min. Under these conditions, the retention time (R_T_) for NMN and nicotinic acid mononucleotide (NaMN) were 4.1 and 3.6 minutes, respectively. The standard reaction medium (1 mL) at 37°C was 0.5 mM NMN and 14 nM purified AtCinA in 50 mM sodium phosphate buffer pH 7.5. At regular intervals during the 15 min that the assay lasted, aliquots were taken, stopped at pH 3.0 with TFA (1% final concentration), kept on ice for 10 min and centrifuged for 10 min at 12000*g* before being injected into the HPLC equipment. One unit of activity was defined as the amount of enzyme required to cleave 1 μmol of NMN releasing 1 μmol of NaMN in 1 min. The above mentioned standard reaction medium was also used to determine the kinetic parameters, but using different NMN concentrations (0–10 mM). Data were obtained in three repeated experiments and fitted by non-linear regression to the Michaelis-Menten equation.

### Stability assays

The pH-stability at 37°C was assayed by measuring the residual activity of AtCinA after incubation in different buffers (50 mM) at different pHs (6.0 to 10.0) in the absence of substrate. In order to maintain the ionic strength, 160 mM acetate buffer pH 5.0 was used. Heat-stability assay was carried out under the same conditions, but incubating the enzyme at pH 7.5 from 20 to 90°C. Aliquots were taken at different times, cooled on ice, and then assayed in the standard reaction medium.

Protein melting curves to determine protein stability were obtained in the presence of the fluorescent dye SYPRO Orange (Molecular Probes). This thermal shift assay was carried out in different buffers (50 mM) containing 10X Sypro Orange (emission at 530 nm and excitation at 490 nm) and 10 μg of purified AtCinA, using a 7500 RT-PCR equipment (Applied Biosystems), as previous described [25].

### Additive effect assay

AtCinA activity was evaluated in the presence of ethylenediaminetetraacetic acid (EDTA), urea, 2-mercaptoethanol, phenylmethanesulfonyl fluoride (PMSF), sodium dodecyl sulfate (SDS), or different ion metals (LiCl, ZnCl_2_, CaCl_2_, MgCl_2_, CoCl_2_, or MnCl_2_). Chemical reagents or metals at a final concentration of 1 mM and 10 mM were incubated with the enzyme in 20 mM HEPES pH 7.5 for 1 hour on ice. Substrate was then added to the reaction mixture and the assay was performed in the standard reaction medium (1 mL) using appropriate blanks as controls. The experiments were carried out in triplicate. Relative activity was reported, considering as 100% the activity in the absence of chemicals or metals.

### *In silico* analysis

The sequences of previously described NMN deamidases were aligned using Clustal Omega [26], and displayed using ESPript [27]. Mutation correlation analysis was made with 2869 sequences, using Mistic (Mutual Information Server To Infer Coevolution) web server (http://mistic.leloir.org.ar) [28]. The Protein-Ligand Interaction Profiler web site (https://projects.biotec.tu-dresden.de/plip-web/plip/) was used for the identification of non-covalent interactions between proteins and their ligands [29]. Protein interactions (ion-pairs, H-bonds, etc) were determined using Protein Interactions Calculator web server (http://pic.mbu.iisc.ernet.in/) [30]. The free energy of dissociation (ΔG^diss^) of AtCinA dimer was calculated in the PISA server (http://www.ebi.ac.uk/pdbe/pisa/) [31]. Protein structures images and structural alignments were obtained with Chimera [32].

## Results

### Amino acid sequence comparison

**Fig 2:**
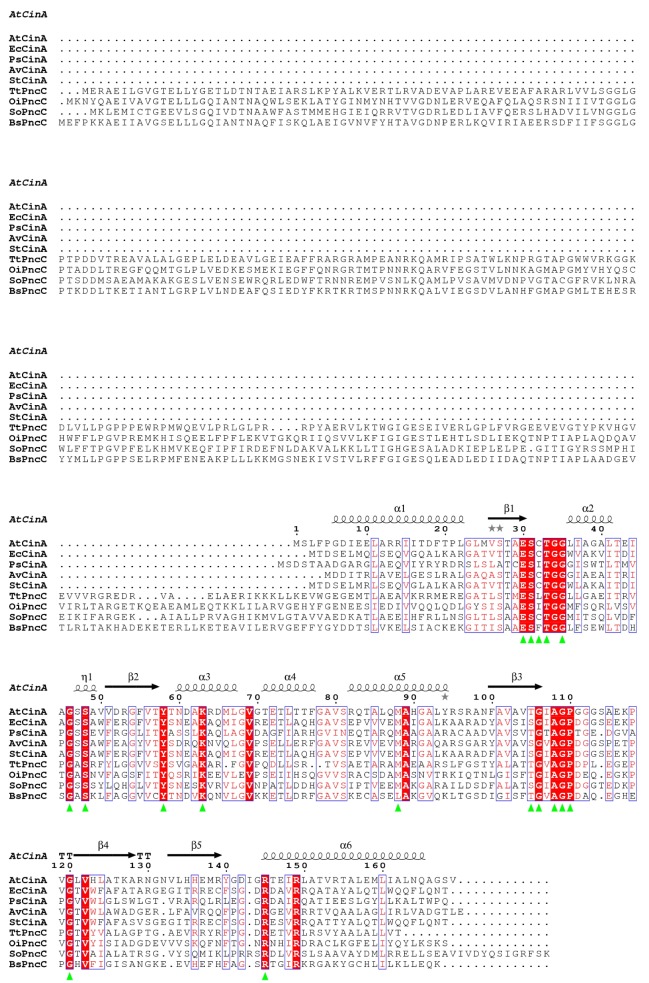
Multiple sequence alignment of *A*. *tumefaciens* nicotinamide mononucleotide deamidase with its homologues. ESPript outputs of CinA after alignment by Clustal Omega with the corresponding sequences from *Agrobacterium tumefaciens* CinA (AtCinA, UniProt code: A9CJ26), *Escherichia coli* CinA (EcCinA, UniProt code: P0A6G3), *Propionibacterium shermanii* CinA (PsCinA, UniProt code: D7GE75), *Azotobacter vinelandii* CinA (AvCinA, UniProt code: C1DSQ5), *Salmonella typhimurium* CinA (StCinA, UniProt code: Q8ZMK4), *Thermus thermophilus* PncC (TtPncC, UniProt code: Q5SHB0), *Oceanobacillus iheyensis* PncC (OiPncC, UniProt code: Q8EQR8), S*hewanella oneidensis* PncC (SoPncC, UniProt code: Q8EK32), and *Bacillus subtilis* PncC (BsPncC, UniProt code: P46323). Residues strictly conserved across NMN deamidase enzymes are highlighted against a red background. The secondary structure corresponding to the crystallized *A*. *tumefaciens* NMN deamidase (2A9S) is shown, where springs represent helices and arrows represent β-strands. The most conserved residues in the CinA family are marked with triangles.

*A*. *tumefaciens* NMN deamidase-encoding protein (AtCinA) was found in the UniProt database (A9CJ26) as a 169 amino acids putative competence/damage inducible protein A (CinA protein). When it was compared with other NMN deamidases described in the bibliography (**Fig 2**), AtCinA showed moderate degree of amino acid sequence identity with proteins from *Salmonella typhimurium* (UniProt entry: Q8ZMK4) [33], *Azotobacter vinelandii* (UniProt entry: C1DSQ5) [20], *Escherichia coli* (UniProt entry: P0A6G3) [23], *Shewanella oneidensis* (UniProt entry: Q8EK32) [18], *Propionibacterium shermanii* (UniProt entry: D7GE75) [21], *Bacillus subtilis* (UniProt entry: P46323) [34], *Thermus thermophilus* (UniProt entry: Q5SHB0) [22] and *Oceanobacillus iheyensis* (UniProt entry: Q8EQR8) [19], with a 53%, 49%, 47%, 41%, 38%, 37%, 35% and 30% sequence identity, respectively.

**Fig 3:**
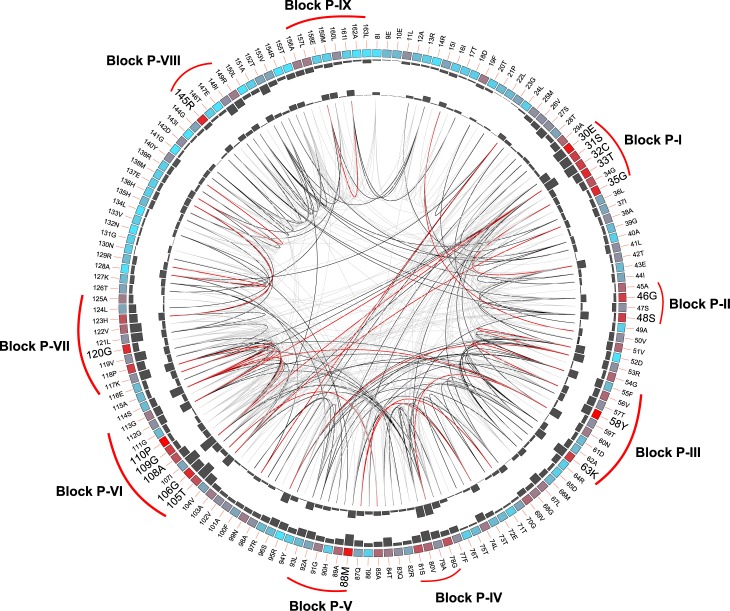
Circos representation of the NMN deamidase family (PF02464). The outer ring shows the amino acid code corresponding to AtCinA (2A9S). Colored square boxes of the second circle indicate the KL (Kullback-Leibler) conservation score (from red to cyan, red: highest; cyan: lowest) [28]. The third and fourth circles show the cMI (cumulative Mutual Information score) and pMI (proximal Mutual Information score) scores as histograms, facing outward and inward, respectively. Lines in the center of the circle connect pairs of positions with MI (Mutational Information) score >6.5. Red lines represent the top 5%; black ones are between 70 and 95%, while grey ones account for the last 70% [28]. Names of the blocks correspond to those previously described [19], whereas the bold letters are the conserved amino acids.

The mutational correlation analysis carried out with the AtCinA sequence, its corresponding Pfam code (PF02464) and its pdb structure (2A9S) in the Mistic server [28] provided a circos representation with the most conserved positions (E30, S31, C32, T33, G35, G46, S48, Y58, K63, M88, T105, G106, A108, G109, P110, G120, and R145) (**Fig 3**, bold letters; **S1 Fig**, red). These positions, also conserved in **Fig 2**(green triangles), indicate that AtCinA has all the described amino acids that form part of the active site [18,19,22,23], including those belonging to the described block P-I (E30, S31, C32, T33, G35) [19] (**Fig 3**; **S1 Fig**, red), of which S31 and C32 have been related with the catalysis and stabilization of the tetrahedral catalytic intermediate, respectively [23]. The backbone amides of G46 and S48 belonging to the described block P-II [19] are involved in hydrogen bonding with O3P and O2P atoms of the substrate/product (NMN/NaMN) phosphate group, whereas Y58 (block P-III) interacts with O1P (**S1 Fig**) [22]. K63 (block P-III) (**Fig 3**; **S1 Fig**) has been described as acting as a general base in both proposed NMN deamidase catalytic mechanisms [22,23]. M88 (block P-V) is the most conserved amino acid identified by mutational correlation analysis, and has been proposed as an important structural element (**S1 Fig**) [19]. T105 and G106 are thought to be involved in the tetrahedral intermediate rearrangement in the catalytic mechanism proposed for TtPncC (**S1 Fig**) [22]. Backbone amides of A108 and G109 interact with O2´ and O3´ hydroxyls of the ribose ring [22], respectively (**S1 Fig**). Furthermore, P110 (block P-VI) and G120 (block P-VII) seem to play a structural role in the loop between β3 and β4 (**Fig 2**; **S1 Fig**). Finally, R145 makes a salt bridge with O1P and O3P atoms of the phosphate group, representing an important energetic contribution to substrate binding (**S1 Fig**) [22].

### Cloning and biochemical characterization of recombinant AtCinA

*cinA* gene from *A*. *tumefaciens* was cloned into pET24b vector and transformed into *E*. *coli* Rosetta 2(DE3). The soluble recombinant protein obtained at 37°C after induction with IPTG was purified in two simple steps, as described in Materials and Methods. Only one band of about 19 kDa was evident when assayed by SDS-PAGE (**S2 Fig**, lane 1). This, together with the data obtained by gel filtration (about 41 kDa) (**S3 Fig**), confirmed the dimeric nature of AtCinA in solution. This dimeric form also agrees with the deposited crystal structure (PDB code: 2A9S).

The purified enzyme was able to fully transform NMN into NaMN, with no apparent inhibition or side products being observed after 1 hour in the tested conditions (10 mM NMN) (**S4 Fig**), an observation of biotechnological relevance for obtaining pure NaMN. In addition, no activity was detected when the enzyme was incubated with NAD^+^, NADH or nicotinamide, even at high enzyme concentrations, as has previously been described for *Shewanella oneidensis* PncC [18]. These results indicate the strict substrate specificity of the enzyme.

**Fig 4:**
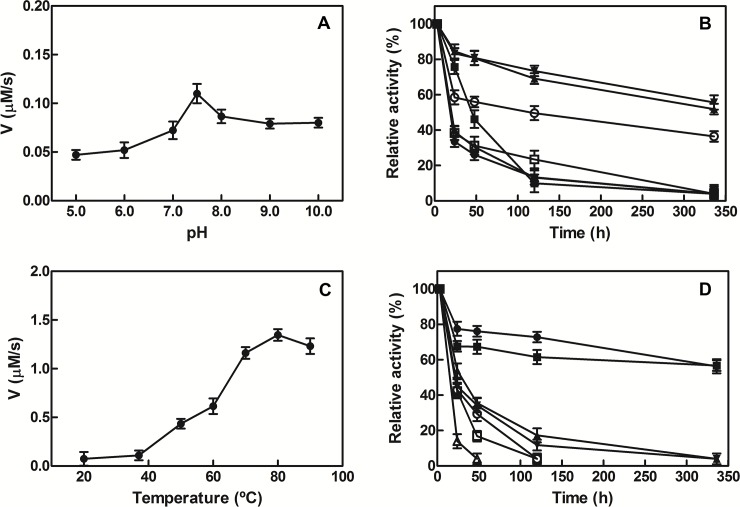
Effect of pH and temperature on AtCinA activity. A) pH profile for AtCinA. The assay conditions at 37°C were 0.5 mM NMN and 14 nM purified AtCinA. The buffers used (50 mM) were sodium phosphate (pH 6.0–7.5), Tris-HCl (pH 8.0) and glycine-NaOH (pH 9.0–10.0). Sodium acetate at pH 5.0 was used at 160 mM in order to maintain the same ionic strength. B) pH-stability. AtCinA was incubated at 37°C for different periods of time at different pHs, and the activity was measured under standard conditions. The buffers used (50 mM) were sodium phosphate pH 6.0 (■), pH 7.0 (▲), pH 7.5 (▼), Tris-HCl pH 8.0 (○), glycine pH 9.0 (∕) and pH 10.0 (**△**), except for sodium acetate pH 5.0 (●), which was at 160 mM as in A). C) Temperature profile for AtCinA activity. Conditions were the same as above, but at different temperatures (20–90°C) in 50 mM sodium phosphate pH 7.5. D) Thermal stability at pH 7.5. AtCinA was incubated for different periods of time at different temperatures [4°C (●), 25°C (■), 35°C (▲), 40°C (▼), 50°C (○), 60°C (∕), and 70°C (ρ)], and the activity was measured in the standard reaction conditions.

The activity of AtCinA was tested under buffered conditions over the pH range of 5.0 to 10.0 (**Fig 4A**). The enzyme displayed activity in conditions that ranged from acidic to basic, with a maximum at pH 7.5. At basic pHs, the enzyme still showed high deamidase activity (~80%), whereas at lower values than pH 7.0 only 42% activity was found (**Fig 4A**). Interestingly, the enzyme was extremely stable at neutral pHs when incubated at 37°C, maintaining above 50% of its activity for 14 days (**Fig 4B**). However, its activity decreased rapidly after 24 hours at both acid and basic pHs, except at pH 8.0, where its half-life was 5 days (**Fig 4B**).

The effect of temperature on the activity of the recombinant enzyme was investigated over a wide range of temperatures (20–90°C). AtCinA showed maximum activity at 80°C, and more than 90% of its activity remained at 90°C (**Fig 4C**). At these high temperatures (80–90°C), no deamidation/decomposition of NMN was observed in the absence of enzyme, when followed by HPLC for at least 3 hours (**S5 Fig**). However, above 80°C, a rapid inactivation of the enzyme was observed in the absence of substrate (**S6 Fig**), although it still showed activity (14%) at 70°C after 24 h (**Fig 4D**). This result contrasts with the low stability described for EcCinA at the same temperature (20% residual activity after 1 h) [23]. At temperatures below 40°C, the enzyme remained relatively stable (half-life of 24 h), but below 25°C the enzyme half-life was greater than 15 days (**Fig 4D**).

**Fig 5:**
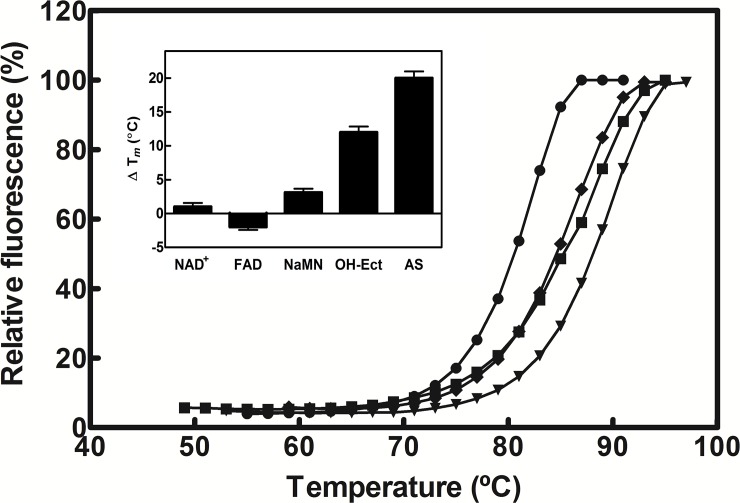
Thermal shift assay of AtCinA. Melting temperature curves of purified enzyme (10 μg) were obtained in the presence of fluorescent probe SYPRO Orange in MilliQ^®^ water (◆), 160 mM sodium acetate pH 5.0 (●), 50 mM sodium phosphate pH 7.5 (▼), and 50 mM glycine pH 10.0 (■). Inset. Effect of different modulators on the melting temperature of AtCinA. Nucleotides were used at 1 mM (NAD^+^, FAD and NaMN), whereas hydroxy-ectoine (OH-Ect) and ammonium sulfate (AS) were used at 2 M and 1.5 M, respectively. Differences in Δ*Tm* were calculated by subtracting the MilliQ^®^ water *Tm* value from the *Tm* values obtained for the enzyme in the different conditions used.

In order to confirm the high pH and temperature stability of AtCinA, a thermal shift assay was carried out (**Fig 5**). Taken the melting temperature (*Tm*) of the enzyme in MilliQ^®^ water as a reference (85.0 ± 0.1°C) (**Fig 5**, diamonds), a similar pH profile as in **Fig 4A** was obtained when Δ*Tm* was plotted against pH (**S7 Fig**), with a maximal *Tm* at pH 7.5 (89.0 ± 0.2°C) (**Fig 5**, triangles) and very similar values were obtained at basic pHs (87.0 ± 0.1°C at pHs 9.0 and 10.0) (**Fig 5**, squares; **S7 Fig**). However, a decrease in *Tm* was observed at pH 5.0 both at 50 mM and 160 mM (80.0 ± 0.1°C) (**Fig 5**, filled circles; **S7 Fig**), reflecting lower stability at acidic pHs. Melting curves were also calculated for AtCinA in the presence of different protein additives. Ammonium sulfate (AS) at 1.5 M was the best stabilizer, increasing *Tm* by about 20°C, followed by 2 M hydroxyectoine, which raised the *Tm* by 12°C (**Fig 5**, inset). However, no protection was found with dinucleotides such as NAD^+^ or FAD (at 1 mM), whereas a slight increase in *Tm* (3.5°C) was observed for NaMN (**Fig 5**, inset).

Finally, the effects of metal ions, common inhibitors and denaturants on AtCinA activity were studied (**S2 Table**). None of the six metal ions tested produced any significant change in activity at either of the concentrations used (1 and 10 mM). In addition, high EDTA concentrations only produced a slight decrease in the activity, suggesting that metal ions are not required. Moderate inhibition (25–30%) of AtCinA activity was observed in the presence of PMSF (**S2 Table**), indicating that AtCinA does not act as a “classic” serine hydrolase. This result is in agreement with that previously described for EcCinA with PMSF [23]. Finally, the chaotropic denaturant urea and 2-mercaptoethanol had no significant effect on the activity, whereas the detergent SDS at 10 mM totally inhibited the enzyme (**S2 Table**). Overall, these results indicate that AtCinA is a stable enzyme and does not require metal ions to express activity.

### Kinetic analysis of NMN deamidation

**Fig 6:**
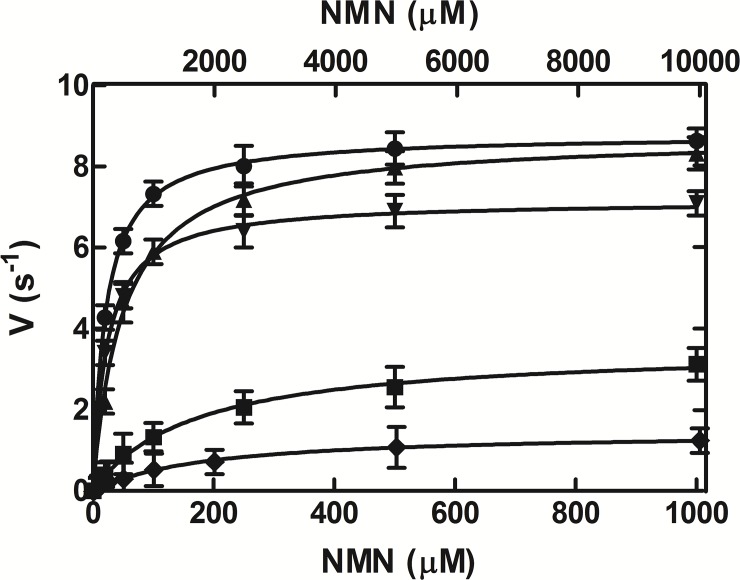
Effect of NMN concentration on AtCinA activity. The activity was measured in the standard reaction conditions in the presence of increasing concentrations of up to 1 mM NMN, except in the case of Y58F mutant, when the concentration ranged from 0 to 10 mM (upper X-scale). Enzyme concentrations were 14 nM for wild type (●), 17 nM for C32 mutant (▼), 14 nM for S48A mutant (▲), 20 nM for T105A mutant (■), and 658 nM for Y58F mutant (◆).

The effect of NMN concentration on the AtCinA reaction rate displayed a Michaelis-Menten kinetic at pH 7.5 and 37°C (**Fig 6,** circles). The K_m_ calculated for NMN was 24 ± 2 μM with a *k*_cat_ of 8.7 ± 0.2 s^-1^ and a *k*_cat_/K_m_ of 362.5 mM^-1^ s^-1^ (**Table 1**). In order to clarify the effect on the kinetics, eight amino acids were selected for site-directed mutagenesis based on previously reported docking [18,19] and structural studies [22]. These key amino acids were seemed to be highly conserved in our coevolutionary relationship analysis between two positions in the CinA family (**Fig 3**). Six mutants (C32A, S48A, Y58F, Y58A, T105A and R145A) were designed to study their implication in substrate binding, and two (S31A and K63) to determine their contribution to catalysis. All mutants were expressed as soluble proteins into *E*. *coli* cells, migrating to the same position as the wild type enzyme (about 19 kDa) in SDS-PAGE (**S2 Fig**). However, only four mutants (C32A, S48A, Y58F and T105) showed activity, but with reduced catalytic efficiency compared to wild type (**Table 1; Fig 6**). Although the S48A mutant showed the same *k*_cat_ as the wild type (**Fig 6)**, its K_m_ value for NMN increased two-fold, with a concomitant reduction in catalytic efficiency (47%) (**Table 1**). However, the C32A mutant displayed a similar K_m_ to the wild type but a slightly reduced *k*_cat_, which resulted in a 28% reduction in catalytic efficiency. This reduction was higher in T105A mutant (94%) due to both an increase in K_m_ (158 ± 15 μM) and a decrease in *k*_cat_ (3.5 ± 0.1 s^-1^) (**Table 1; Fig 6**). Finally, the catalytic efficiency of Y58F mutant was reduced by up to 99.7%, since its K_m_ increased by two orders of magnitude (1930 ± 50 μM) and its *k*_cat_ decreased 5.8-fold (1.5 ± 0.1 s^-1^) (**Table 1; Fig 6**).

**Table 1:** Kinetic parameters and melting temperature (*Tm*) of wild-type AtCinA and its mutants.

	K_m_ (μM)	*k*_*cat*_ (s^-1^)	*k*_*cat*_ /K_m_ (mM^-1^s^-1^)	*Tm* (°C)^†^
**Wild-type**	24 ± 2	8.7 ± 0.2	362.5	85 ± 0.1
**S31A**	-	ND^*^	-	86 ± 0.2
**C32A**	28 ± 3	7.3 ± 0.2	260.0	83 ± 0.2
**S48A**	51 ± 3	8.7 ± 0.3	170.6	83 ± 0.1
**Y58A**	-	ND^*^	-	83 ± 0.2
**Y58F**	1930 ± 50	1.5 ± 0.1	0.8	83 ± 0.2
**K63A**	-	ND^*^	-	78 ± 0.1
**T105A**	158 ± 14	3.5 ± 0.1	22.1	81 ± 0.1
**R145A**	-	ND^*^	-	54 ± 0.1

^†^ *Tm* values were obtained in MilliQ^®^ water.

^*^ ND, not detectable.

**Fig 7:**
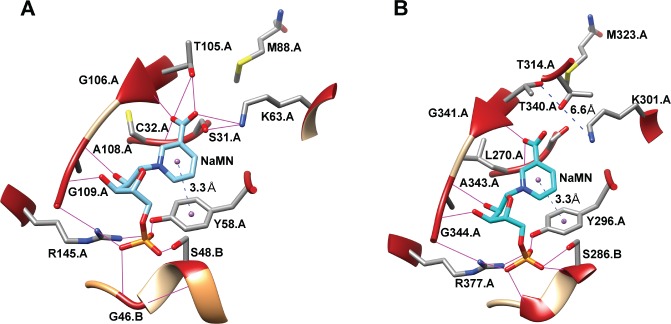
NaMN binding site. A) AtCinA binding site. NaMN (light cyan) was placed in the AtCinA structure (2A9S) using a structural alignment with the corresponding TtPncC structure (4UOC) [22] with Chimera [32]. B) TtPncC binding site with crystalized NaMN (cyan) (4UOC) [22]. Hydrogen bonds are shown in magenta. Distances are displayed as dashed lines.

To ascertain whether or not the absence of activity in S31A, Y58A, K63A and R145A was due to changes in their structure, the effect of each mutation on *Tm* was studied (**Table 1**; **S8 Fig**). All mutants tested showed similar melting temperatures as wild type (about 80°C), except for R145A, whose *Tm* decreased to 54°C (**Table 1**; **S8 Fig**). This low value indicates a less structured enzyme, with a possible altered NMN binding site, as previously described for such mutant in EcCinA [23]. Of note was the absence of activity in Y58A compared with Y58F, despite both having the same *Tm*. This could be related to the presence of an aromatic ring in Y58F (but not in Y58A), which could interact with the nicotinamide ring, helping the substrate to bind to the active center, even in the absence of the important hydrogen bond displayed between Y58 and NaMN phosphate group in the crystal structure of TtPncC (pdb code 4UOC) (**Fig 7**) [22]. Finally, S31A and K63A mutants completely lost their catalytic activity, which confirmed that these amino acids are associated with catalysis [18,23].

## Discussion

NMN deamidases have been phylogenetically classified into two large groups and 7 subgroups based on their sequences and structural information on protein databases [19]. Among them, NMN deamidase from plant pathogen *A*. *tumefaciens* is included in subgroup 2.2.3 [19]. This subgroup basically shows one-domain enzymes (CinAs). Although most microorganisms belonging to this group are proteobacteria, the group also includes important pathogens such as *Brucella abortus*, *Helicobacter pylori* or *Mycoplasma pneumoniae*. However, no NMN deamidase from this group or from alpha-proteobacteria has been characterized to date. Curiously, the only information available for this subgroup is the AtCinA structure (2A9S), deposited by Midwest Center for Structural Genomics in the Protein Data Bank, but not published.

### AtCinA shows a broad range of activity from neutral to basic pH

The biochemical studies carried out after the cloning and purification of AtCinA showed it to be the second CinA, after *A*. *vinelandii* CinA [20], to have a neutral optimum pH (pH 7.5) (**Fig 4A**). However, AtCinA is more active at basic pHs than *A*. *vinelandii* CinA with almost double the activity at pH 8.5 (82% *vs* 46%) [20]. This neutral optimum pH contrasts with the values described for other CinAs, such as *E*. *coli* CinA (EcCinA) (pH 9.0) [13], *Salmonella typhimurium* CinA (pH 8.5–8.7) [15,35] and, especially, *Propionibacterium shermanii* CinA (pH 5.6) [21]. Of note is the fact that, AtCinA showed the same broad range of activity from neutral to basic pHs (pH 6.5–10.0) as displayed by the two-domain NMN deamidases (PncCs), such as those of *Oceanobacillus iheyensis* PncC (OiPncC) (pH 7.0–10.0) [19] and *Shewanella oneidensis* PncC (SoPncC) (pH 5.5–9.0) [18]. It also showed the same high residual activity at pH 10.0 (about 80%) (**Fig 4A**). Importantly, the pH-stability study (**Fig 4B**) revealed that AtCinA is a very stable enzyme at pH 7.0 after 24 h, showing 84% residual activity, which is the highest stability reported to date, since OiPncC displayed no activity after the same time [19].

### AtCinA has a high melting temperature

The study of the effect of temperature (**Fig 4C**) on AtCinA activity revealed it to be the NMN deamidase with the highest optimum temperature (80°C) described in the bibliography. This value is about 15°C above that described for OiPncC (65°C) [19] and *A*. *vinelandii* CinA (64°C) [20]. However, its stability at high temperatures was low in the absence of substrate, as previously observed for OiPncC [19], but it is still higher than the latter enzyme. For example, at 50°C, AtCinA showed 43% activity (**Fig 4D**) compared with the 6% described for OiPncC [19]. Thus, when the best pH and temperature were used together (4–25°C and pH 7.0–7.5), the enzyme showed the highest stability described for NMN deamidases, maintaining 55% of its activity after 14 days (**Fig 4D**). This high stability was corroborated by the thermal shift assay carried out (**Fig 5**), where a similar pH profile as in **Fig 4A** was obtained (**S7 Fig**) with a clear maximum at pH 7.5 (89 ± 0.1°C), a slight decrease in stability at basic pHs and a reduction at acidic pHs. These data are the first values of a melting temperature reported for a NMN deamidase. In order to explain this high melting temperature, the AtCinA structure was compared with that of the CinA domain of the bifunctional NMN deamidase from *Thermus thermophilus* (PDB 4UOC). The AtCinA dimer showed a similar number of hydrogen bonds per residue than the CinA domain of TtPncC (3.76 vs 3.66), and a surprisingly high number of ion pairs (26 vs 19). In fact, the number of ion pairs per residue in AtCinA was 0.056, which is higher than the average value (0.04) found in proteins [36]. Most of these ion pairs are engaged in stabilizing helices through intra or inter-helix interactions. Furthermore, charged residues compensate most of the N- and C-terminus α-helix macrodipoles, a characteristic that could contribute to thermoestabilization [36,37]. The number of charged residues is similar in both enzymes (20% vs 21%) [38] and the Arg/(Arg+Lys) ratio, which normally increases in thermophilic enzymes is very similar (0.8 vs 0.88). Another important aspect to explain the stability at high temperature is that AtCinA appears to be a very compact and thermodynamically stable dimer according to its positive free energy of dissociation (ΔG^diss^ = 23 Kcal/mol), as calculated by the PISA server [31]. AtCinA also shows a large dimeric interface area, similar to that found in TtPncC (19% vs 15% of the total accessible area of the monomer). In addition, this dimeric interface area includes several stabilizing ion pairs between the dimer subunits. Thus, AtCinA shows many structural characteristics that are very close to those of thermophilic enzymes. In fact, the melting temperature of AtCinA was also higher (between 23–35°C) than those described by our group for other enzymes, such as nicotinamidases [39] or sialic acid aldolases [40], indicating its high stability. In addition, its long-term stability for possible commercial use could be improved by using protein stabilizers, the best found being to be ammonium sulfate, followed by hydroxyectoine (**Fig 5, inset**).

### AtCinA has high catalytic efficiency

As regards kinetic parameters, AtCinA showed a similar K_m_ value for NMN (24 μM) to those described in the bibliography, being close to those of *E*. *coli* CinA and *P*. *shermanii* CinA (6–7 μM) [13,21], but lower than those of OiPncC (180 μM) [19] and *A*. *vinelandii* CinA (1 mM) [20]. Its *k*_cat_ (8.7 s^-1^) was the highest determined, far from that of OiPncC (0.38 s^-1^) [19] and double those of SoPncC (3.3 s^-1^) [18] and EcCinA (4.1 s^-1^) [13]. Thus, it showed a high catalytic efficiency (*k*_cat_/K_m_), which was similar to the best described for EcCinA [23]. However, AtCinA thermal stability was higher than that described for EcCinA [23], which lost its activity in 1 h at 70°C, whereas AtCinA preserved 61% activity under the same conditions (**Fig 4D**).

### Mutational analysis of AtCinA supports the Ser/Lys catalytic dyad mechanism

The mutational analysis carried out showed that the conserved amino acids predicted by Mistic can be used to define not only the binding site or the catalytic amino acids, but also some other important amino acids to preserve the integrity of the binding site. Of the 8 mutants (S31A, C32A, S48A, Y58F, Y58A, K63A, T105, and R145A), only four were active (**Table 1**), and among them, three have never been described (C32A, S48A and Y58F) (**Table 1**). No activity was found for S31A, K63A or R145A mutants, since these residues are important for catalysis (S31 and K63) and for phosphate binding (R145), respectively. In fact, binding of the NMN phosphate moiety is crucial for fixing the substrate in the binding site, since four hydrogen bonds are formed, two with R145, one with Y58 and one with S48, although the last residue corresponds to the adjacent subunit in the AtCinA dimer (**Fig 7A; S1 Fig**). The importance of this network of hydrogen bonds (**Fig 7A**, magenta) was evident from the 2.1-fold decrease in the catalytic efficiency of the S48A mutant compared with wild type enzyme. This decrease in catalytic efficiency was less pronounced in C32A (only 1.4-fold), since the substitution of a cysteine by an alanine does not much alter the orientation of the nicotinyl carbonyl oxygen, especially when another amino acid such as G106 is also involved [23]. However, this effect is more marked in T105A with a 16-fold decrease caused by an impairment of both K_m_ (6.5-fold increase) and *k*_cat_ (2.5-fold decrease). This result illustrates the important contribution of T105 to the correct binding and orientation of the NMN amide group for catalysis, as has previously been described for a similar mutant in EcCinA (S103A) [23]. Finally, the most dramatic decrease in catalytic efficiency was observed in Y58F (460-fold), produced by a large increase in K_m_ and a 6-fold decrease in *k*_cat_. However, this replacement of tyrosine by phenylalanine still preserved activity, while its replacement by alanine (Y58A) completely abolished it (**Table 1**). This effect, never before described, is not related with a general change in structure, since both mutants have similar *Tm* values (83°C) (**Table 1**). Rather, it might be due to aromatic π-stacking between the nicotinamide moiety of NMN/NaMN and tyrosine/phenylalanine aromatic ring, as predicted by the Protein-Ligand Interaction Profiler web site [29], not only for the NaMN structurally aligned to the crystal structure of AtCinA (**Fig 7A**), but also for that crystallized in TtPncC (PDB code 4UOC) [22] (**Fig 7B**), with an average distance of 3.3 Å between the centroids of the aromatic rings in both structures, as calculated by Chimera (**Fig 7**) [32].

This mutational analysis also pointed to the total loss of activity in R145A but, in this case, the changes induced in the structure seemed to be responsible for the loss in activity, as confirmed by the 31°C decrease in *Tm* compared with that of wild type enzyme (**Table 1**). This result agrees with that previously described by Raffaelli´s group for the equivalent R142A mutant in *E*. *coli* using circular dichroism [23]. These authors found a different fingerprint for the side chains in the near-UV spectrum compared with that of wild type, and concluded that R142A is less structured [23]. By contrast, a well-structured near-UV spectrum was found in Y56A (Y58A in AtCinA) [23], which also agrees with the result found in the thermal shift assay carried out with AtCinA Y58A mutant (**Table 1**). However, the absence of activity in S31A and K63A mutants, with no apparently important changes in their structures according to the similar *Tm* values obtained compared with that of wild type (**Table 1**), reinforces the Ser/Lys catalytic dyad mechanism proposed by Raffaelli´s group [23], in which, K63 acts a general base activating S31 for the nucleophilic attack of the amide bond of the nicotinamide moiety, giving rise to the oxyanion tetrahedral intermediate stabilized by main chain amides of C32 and G106 (**Fig 7A**). By contrasts, in the asparaginase II-like catalytic mechanism proposed by Derrick´s group [22], a water molecule activated by K301 (K63 in AtCinA) produced the nucleophilic attack on the amide bond, and then, the oxyanion tetrahedral intermediate formed with amino acids L270 and G431 was rearranged with the aid of T340 (T105 in AtCinA) and K301 [22]. However, these two latter amino acids are separated in the TtPncC crystal structure by 6.6 Å (**Fig 7B**). This distance, together with the fact that T340 is not strictly conserved in the CinA family, where it is replaced by a serine (e.g. S103 in EcCinA) in half of the family [23] and the fact that AtCinA T105A mutant still preserves activity (**Table 1**; **Fig 6**), make the proposed asparaginase II-like catalytic mechanism less likely.

## Conclusions

The NMN deamidase from *Agrobacterium tumefaciens* was cloned in *E*. *coli*, overexpressed, and purified to obtain a stable 41-kDa homodimeric protein. The enzyme was able to hydrolyze NMN with a catalytic efficiency similar to the best efficiencies found for NMN deamidases, but with higher thermal and pH stabilities. These relevant operational characteristics open up the possibility of developing a cost-effective enzymatic process for obtaining NaMN, which along with other NAD^+^ analogs and related mononucleotides, is now considered a promising potential therapeutic agent. Finally, the mutational analysis carried out supported the classification of AtCinA in the Ser/Lys amidohydrolase family. However, further structural and biochemical research is needed to confirm this catalytic mechanism of NMN deamidase activity in other members of the CinA family.

## Supporting information

S1 Fig AtCinA structure. Both subunits of crystallized AtCinA (2A9S) are shown as ribbons (subunit A, sandy brown; subunit B, rosy brown). The conserved amino acids found in MISTIC server [28] for NMN deamidases (PF02464) are shown in red. NaMN is shown in cyan after structural alignment with TtPncC (4UOC) [22] using Chimera [32].
(PDF)

S2 Fig SDS-PAGE of purified AtCinA and its mutants. 10 μg of AtCinA and its corresponding mutants obtained after the HisTrap column step. M: molecular weight standards (Fisher Scientific: BP3603). Lane 1, AtCinA wild type; lane 2, S31A; lane 3, C32A; lane 4, S48A; lane 5, Y58A; lane 6, Y58F; lane 7, K63A; lane 8, T105A; and lane 9, R145A.
(PDF)

S3 Fig Size-exclusion chromatography of AtCinA. The bottom panel illustrates the optical absorption (280 nm) of the effluent. The top panel illustrates a semi-logarithmic plot of molecular mass *vs*. elution volume. Black circles correspond to the following protein standards (mass is given in kDa in parentheses): cytochrome c (12.4), carbonic anhydrase (29), BSA (66.4), alcohol dehydrogenase (150), and β-amylase (200). The red circle plot corresponds to the elution volume of the main peak against the sequence-deduced mass for a dimer (41 kDa) of AtCinA.
(PDF)

S4 Fig Time-course of NMN conversion into NaMN catalyzed by AtCinA. The reaction medium at 37 °C contained 10 mM of NMN and 140 nM AtCinA in 50 mM sodium phosphate buffer pH 7.5. (●) NMN and (○) NaMN.
(PDF)

S5 Fig Thermal stability of NMN at 85°C. Dashed black line corresponds to NMN at zero time, and solid red line corresponds to NMN after 3 hours. No deamidation/decomposition was observed during this period.
(PDF)

S6 Fig Thermal stability of AtCinA at 80°C. AtCinA was incubated for different periods of time at 80°C, and the activity was measured in the standard reaction conditions.
(PDF)

S7 Fig Effect of different pHs on the melting temperature of AtCinA. Differences in Δ*Tm* were calculated by subtracting the MilliQ^®^ water *Tm* value from the *Tm* values obtained for the enzyme at different pHs (50 mM sodium acetate pH 5.0, 50 mM sodium phosphate pHs 6.0–7.5, 50 mM Tris-HCl pH 8.0, and 50 mM glycine-NaOH pH 9.0–10.0). pH 5.0^*^ indicates that the experiment was also carried out at 160 mM sodium acetate pH 5.0.
(PDF)

S8 Fig Thermal shift assay of AtCinA and its mutants. Melting temperature curves of purified enzyme (10 μg) were obtained in the presence of fluorescent probe SYPRO Orange. Wild type (●), S31A (○), C32A (△), S48A (■), Y58A (▲), Y58F (▼), K63A (◆), T105A (▽), and R145A (∕).
(PDF)

S1 Table Sequences of primers used to clone AtCinA and its corresponding mutants. (PDF)

S2 Table Effects of metal ions and chemicals on AtCinA activity. (PDF)

## References

[1] Butepage M, Eckei L, Verheugd P, Luscher B (2015) Intracellular Mono-ADP-Ribosylation in Signaling and Disease. Cells 4: 569–595. PubMed Central PMCID: PMC4695847. doi: 10.3390/cells4040569 26426055 10.3390/cells4040569 PMC4695847

[2] Verheugd P, Butepage M, Eckei L, Luscher B (2016) Players in ADP-ribosylation: readers and erasers. Curr Protein Pept Sci 17: 654–667. 27090904 10.2174/1389203717666160419144846

[3] Yang Y, Sauve AA (2016) NAD+ metabolism: Bioenergetics, signaling and manipulation for therapy. Biochim Biophys Acta. 10.1016/j.bbapap.2016.06.014 PMC5521000 27374990

[4] Kupis W, Palyga J, Tomal E, Niewiadomska E (2016) The role of sirtuins in cellular homeostasis. J Physiol Biochem 72: 371–380. PubMed Central PMCID: PMC4992043. doi: 10.1007/s13105-016-0492-6 27154583 10.1007/s13105-016-0492-6 PMC4992043

[5] Sorci L, Ruggieri S, Raffaelli N (2014) NAD homeostasis in the bacterial response to DNA/RNA damage. DNA Repair (Amst) 23: 17–26. 25127744 10.1016/j.dnarep.2014.07.014

[6] Giblin W, Skinner ME, Lombard DB (2014) Sirtuins: guardians of mammalian healthspan. Trends Genet 30: 271–286. PubMed Central PMCID: PMC4077918. doi: 10.1016/j.tig.2014.04.007 24877878 10.1016/j.tig.2014.04.007 PMC4077918

[7] Nikiforov A, Kulikova V, Ziegler M (2015) The human NAD metabolome: Functions, metabolism and compartmentalization. Crit Rev Biochem Mol Biol 50: 284–297. PubMed Central PMCID: PMC4673589. doi: 10.3109/10409238.2015.1028612 25837229 10.3109/10409238.2015.1028612 PMC4673589

[8] Canto C, Menzies KJ, Auwerx J (2015) NAD(+) Metabolism and the Control of Energy Homeostasis: A Balancing Act between Mitochondria and the Nucleus. Cell Metab 22: 31–53. PubMed Central PMCID: PMC4487780. doi: 10.1016/j.cmet.2015.05.023 26118927 10.1016/j.cmet.2015.05.023 PMC4487780

[9] Opitz CA, Heiland I (2015) Dynamics of NAD-metabolism: everything but constant. Biochem Soc Trans 43: 1127–1132. doi: 10.1042/BST20150133 26614649 10.1042/BST20150133

[10] Ruggieri S, Orsomando G, Sorci L, Raffaelli N (2015) Regulation of NAD biosynthetic enzymes modulates NAD-sensing processes to shape mammalian cell physiology under varying biological cues. Biochim Biophys Acta 1854: 1138–1149. doi: 10.1016/j.bbapap.2015.02.021 25770681 10.1016/j.bbapap.2015.02.021

[11] Covington JD, Bajpeyi S (2016) The sirtuins: Markers of metabolic health. Mol Nutr Food Res 60: 79–91. doi: 10.1002/mnfr.201500340 26463981 10.1002/mnfr.201500340

[12] Park UE, Olivera BM, Hughes KT, Roth JR, Hillyard DR (1989) DNA ligase and the pyridine nucleotide cycle in Salmonella typhimurium. J Bacteriol 171: 2173–2180. PubMed Central PMCID: PMC209874. 2649488 10.1128/jb.171.4.2173-2180.1989 PMC209874

[13] Hillyard D, Rechsteiner M, Manlapaz-Ramos P, Imperial JS, Cruz LJ, et al (1981) The pyridine nucleotide cycle. Studies in Escherichia coli and the human cell line D98/AH2. J Biol Chem 256: 8491–8497. 7021549

[14] Foster JW, Baskowsky-Foster AM (1980) Pyridine nucleotide cycle of Salmonella typhimurium: in vivo recycling of nicotinamide adenine dinucleotide. J Bacteriol 142: 1032–1035. PubMed Central PMCID: PMC294135. 6445894 10.1128/jb.142.3.1032-1035.1980 PMC294135

[15] Kinney DM, Foster JW, Moat AG (1979) Pyridine nucleotide cycle of Salmonella typhimurium: in vitro demonstration of nicotinamide mononucleotide deamidase and characterization of pnuA mutants defective in nicotinamide mononucleotide transport. J Bacteriol 140: 607–611. PubMed Central PMCID: PMCPMC216688. 387742 10.1128/jb.140.2.607-611.1979 PMC216688

[16] Lespinet O, Labedan B (2005) Orphan enzymes? Science 307: 42. 10.1126/science.307.5706.42a 15637255

[17] Fyfe JA, Friedmann HC (1969) Vitamin B 12 biosynthesis. Enzyme studies on the formation of the alpha-glycosidic nucleotide precursor. J Biol Chem 244: 1659–1666. 4238408

[18] Galeazzi L, Bocci P, Amici A, Brunetti L, Ruggieri S, et al (2011) Identification of nicotinamide mononucleotide deamidase of the bacterial pyridine nucleotide cycle reveals a novel broadly conserved amidohydrolase family. J Biol Chem 286: 40365–40375. PubMed Central PMCID: PMC3220592. doi: 10.1074/jbc.M111.275818 21953451 10.1074/jbc.M111.275818 PMC3220592

[19] Sanchez-Carron G, Martinez-Monino AB, Sola-Carvajal A, Takami H, Garcia-Carmona F, et al (2013) New insights into the phylogeny and molecular classification of nicotinamide mononucleotide deamidases. PLoS One 8: e82705 PubMed Central PMCID: PMC3855486. doi: 10.1371/journal.pone.0082705 24340054 10.1371/journal.pone.0082705 PMC3855486

[20] Imai T (1973) Purification and properties of nicotinamide mononucleotide amidohydrolase from Azotobacter vinelandii. J Biochem 73: 139–153. 4144084

[21] Friedmann HC, Garstki C (1973) The pyridine nucleotide cycle: presence of a nicotinamide mononucleotide-specific amidohydrolase in Propionibacterium shermanii. Biochem Biophys Res Commun 50: 54–58. 4683625 10.1016/0006-291x(73)91062-0

[22] Karuppiah V, Thistlethwaite A, Dajani R, Warwicker J, Derrick JP (2014) Structure and mechanism of the bifunctional CinA enzyme from Thermus thermophilus. J Biol Chem 289: 33187–33197. PubMed Central PMCID: PMCPMC4246079. doi: 10.1074/jbc.M114.608448 25313401 10.1074/jbc.M114.608448 PMC4246079

[23] Sorci L, Brunetti L, Cialabrini L, Mazzola F, Kazanov MD, et al (2014) Characterization of bacterial NMN deamidase as a Ser/Lys hydrolase expands diversity of serine amidohydrolases. FEBS Lett 588: 1016–1023. doi: 10.1016/j.febslet.2014.01.063 24530526 10.1016/j.febslet.2014.01.063

[24] Gesto DS, Cerqueira NM, Fernandes PA, Ramos MJ (2013) Unraveling the enigmatic mechanism of L-asparaginase II with QM/QM calculations. J Am Chem Soc 135: 7146–7158. doi: 10.1021/ja310165u 23544711 10.1021/ja310165u

[25] Sanchez-Carron G, Garcia-Garcia MI, Lopez-Rodriguez AB, Jimenez-Garcia S, Sola-Carvajal A, et al (2011) Molecular characterization of a novel N-acetylneuraminate lyase from Lactobacillus plantarum WCFS1. Appl Environ Microbiol 77: 2471–2478. PubMed Central PMCID: PMC3067430. doi: 10.1128/AEM.02927-10 21317263 10.1128/AEM.02927-10 PMC3067430

[26] Sievers F, Higgins DG (2014) Clustal Omega, accurate alignment of very large numbers of sequences. Methods Mol Biol 1079: 105–116. doi: 10.1007/978-1-62703-646-7_6 24170397 10.1007/978-1-62703-646-7_6

[27] Gouet P, Courcelle E, Stuart DI, Metoz F (1999) ESPript: analysis of multiple sequence alignments in PostScript. Bioinformatics 15: 305–308. 10320398 10.1093/bioinformatics/15.4.305

[28] Simonetti FL, Teppa E, Chernomoretz A, Nielsen M, Marino Buslje C (2013) MISTIC: Mutual information server to infer coevolution. Nucleic Acids Res 41: W8–14. PubMed Central PMCID: PMC3692073. doi: 10.1093/nar/gkt427 23716641 10.1093/nar/gkt427 PMC3692073

[29] Salentin S, Schreiber S, Haupt VJ, Adasme MF, Schroeder M (2015) PLIP: fully automated protein-ligand interaction profiler. Nucleic Acids Res 43: W443–447. PubMed Central PMCID: PMC4489249. doi: 10.1093/nar/gkv315 25873628 10.1093/nar/gkv315 PMC4489249

[30] Tina KG, Bhadra R, Srinivasan N (2007) PIC: Protein Interactions Calculator. Nucleic Acids Res 35: W473–476. PubMed Central PMCID: PMC1933215. doi: 10.1093/nar/gkm423 17584791 10.1093/nar/gkm423 PMC1933215

[31] Krissinel E, Henrick K (2007) Inference of macromolecular assemblies from crystalline state. J Mol Biol 372: 774–797. doi: 10.1016/j.jmb.2007.05.022 17681537 10.1016/j.jmb.2007.05.022

[32] Pettersen EF, Goddard TD, Huang CC, Couch GS, Greenblatt DM, et al (2004) UCSF Chimera—a visualization system for exploratory research and analysis. J Comput Chem 25: 1605–1612. doi: 10.1002/jcc.20084 15264254 10.1002/jcc.20084

[33] Foster JW, Kinney DM, Moat AG (1979) Pyridine nucleotide cycle of Salmonella typhimurium: isolation and characterization of pncA, pncB, and pncC mutants and utilization of exogenous nicotinamide adenine dinucleotide. J Bacteriol 137: 1165–1175. PubMed Central PMCID: PMCPMC218297. 220211 10.1128/jb.137.3.1165-1175.1979 PMC218297

[34] Kaimer C, Graumann PL (2010) Bacillus subtilis CinA is a stationary phase-induced protein that localizes to the nucleoid and plays a minor role in competent cells. Arch Microbiol 192: 549–557. doi: 10.1007/s00203-010-0583-7 20480359 10.1007/s00203-010-0583-7

[35] Cheng W, Roth J (1995) Isolation of NAD cycle mutants defective in nicotinamide mononucleotide deamidase in Salmonella typhimurium. J Bacteriol 177: 6711–6717. PubMed Central PMCID: PMCPMC177533. 7592458 10.1128/jb.177.23.6711-6717.1995 PMC177533

[36] Auerbach G, Huber R, Grattinger M, Zaiss K, Schurig H, et al (1997) Closed structure of phosphoglycerate kinase from Thermotoga maritima reveals the catalytic mechanism and determinants of thermal stability. Structure 5: 1475–1483. 9384563 10.1016/s0969-2126(97)00297-9

[37] Robinson CR, Sligar SG (1993) Electrostatic stabilization in four-helix bundle proteins. Protein science: a publication of the Protein Society 2: 826–837. PubMed Central PMCID: PMC2142490. 8388289 10.1002/pro.5560020512 PMC2142490

[38] Imanaka T (2011) Molecular bases of thermophily in hyperthermophiles. Proceedings of the Japan Academy Series B, Physical and biological sciences 87: 587–602. PubMed Central PMCID: PMC3309922. doi: 10.2183/pjab.87.587 22075760 10.2183/pjab.87.587 PMC3309922

[39] Sanchez-Carron G, Garcia-Garcia MI, Zapata-Perez R, Takami H, Garcia-Carmona F, et al (2013) Biochemical and mutational analysis of a novel nicotinamidase from Oceanobacillus iheyensis HTE831. PLoS One 8: e56727 PubMed Central PMCID: PMC3581539. doi: 10.1371/journal.pone.0056727 23451075 10.1371/journal.pone.0056727 PMC3581539

[40] Garcia-Garcia MI, Gil-Ortiz F, Garcia-Carmona F, Sanchez-Ferrer A (2014) First functional and mutational analysis of group 3 N-acetylneuraminate lyases from Lactobacillus antri and Lactobacillus sakei 23K. PLoS One 9: e96976 PubMed Central PMCID: PMC4016182. doi: 10.1371/journal.pone.0096976 24817128 10.1371/journal.pone.0096976 PMC4016182

