# Supplementary figures and images for: Characterization and mutational analysis of a nicotinamide mononucleotide deamidase from Agrobacterium tumefaciens showing high thermal stability and catalytic efficiency
Source: PLoS One. 2017 Apr 7;12(4):e0174759. doi: 10.1371/journal.pone.0174759 (PMC5384747; doi:10.1371/journal.pone.0174759)

**Subunit A**

**Subunit B**

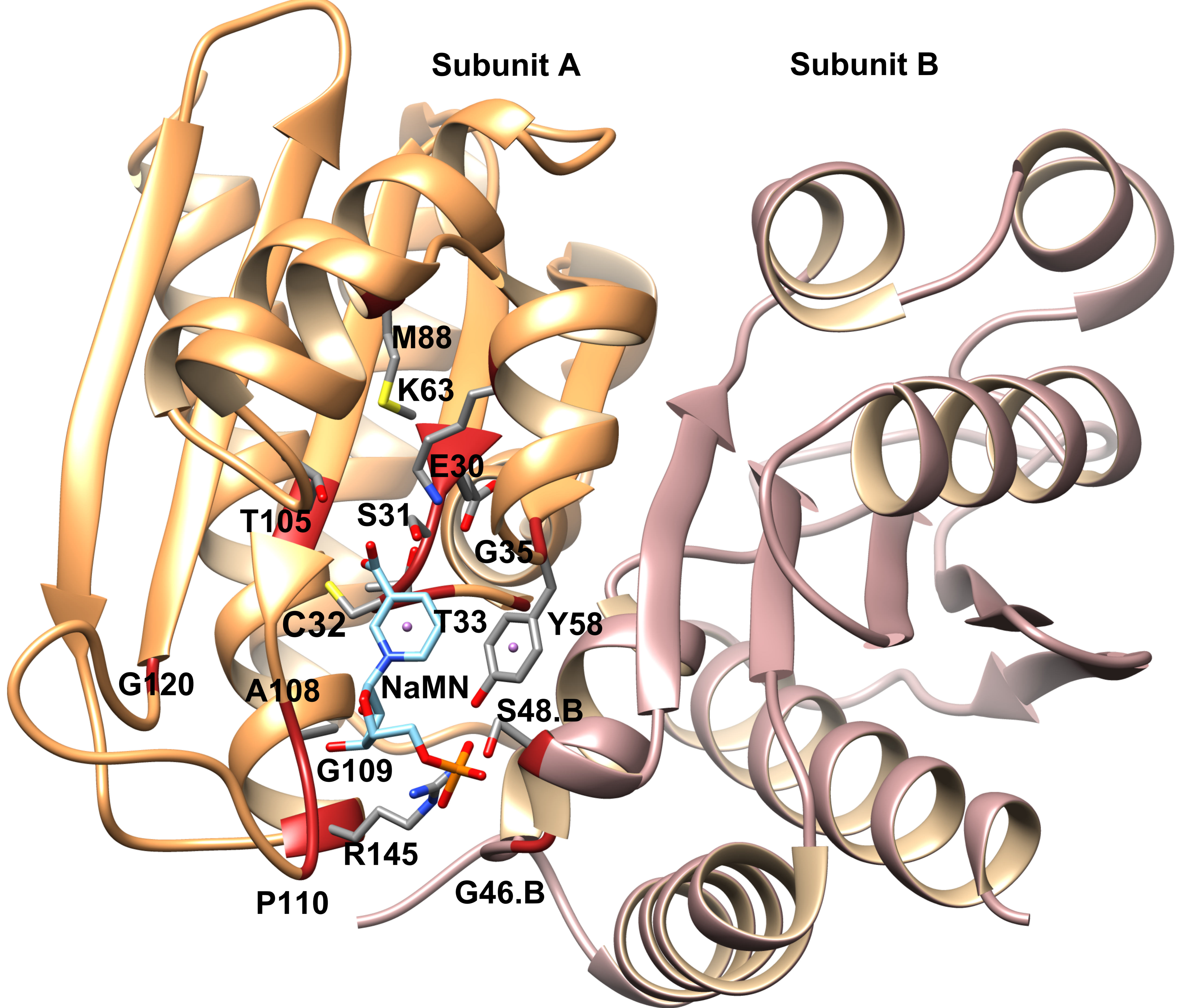

Supplement: S1 Fig — Both subunits of crystallized AtCinA (2A9S) are shown as ribbons (subunit A, sandy brown; subunit B, rosy brown). The conserved amino acids found in MISTIC server [28] for NMN deamidases (PF02464) are shown in red. NaMN is shown in cyan after structural alignment with TtPncC (4UOC) [22] using Chimera [32]. (PDF) [file pone.0174759.s001.pdf]

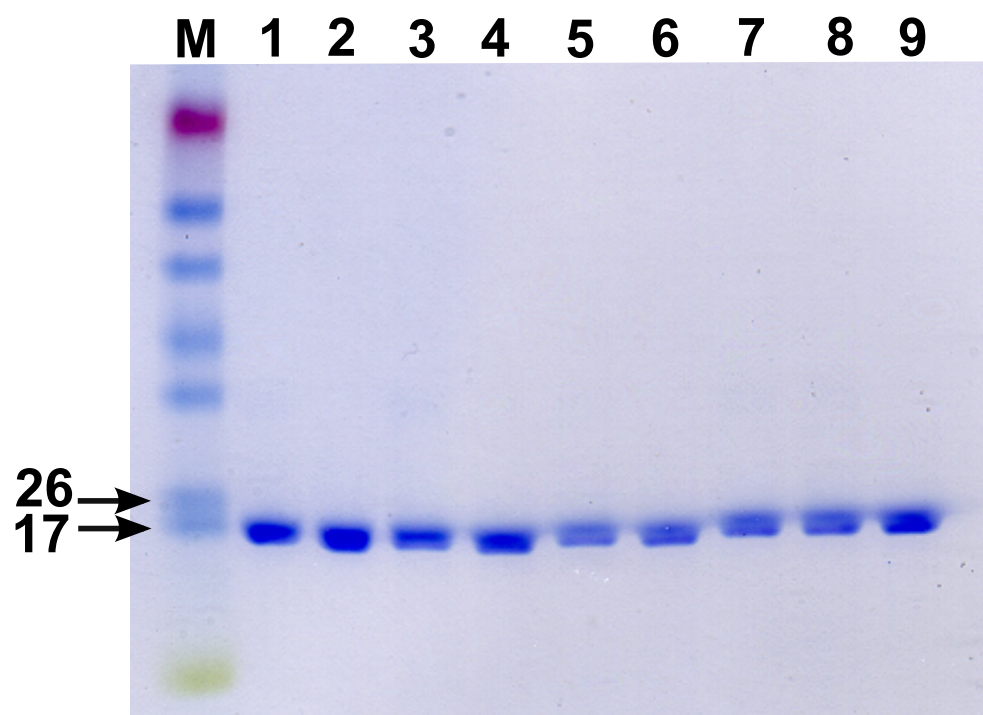

Supplement: S2 Fig — 10 μg of AtCinA and its corresponding mutants obtained after the HisTrap column step. M: molecular weight standards (Fisher Scientific: BP3603). Lane 1, AtCinA wild type; lane 2, S31A; lane 3, C32A; lane 4, S48A; lane 5, Y58A; lane 6, Y58F; lane 7, K63A; lane 8, T105A; and lane 9, R145A. (PDF) [file pone.0174759.s002.pdf]

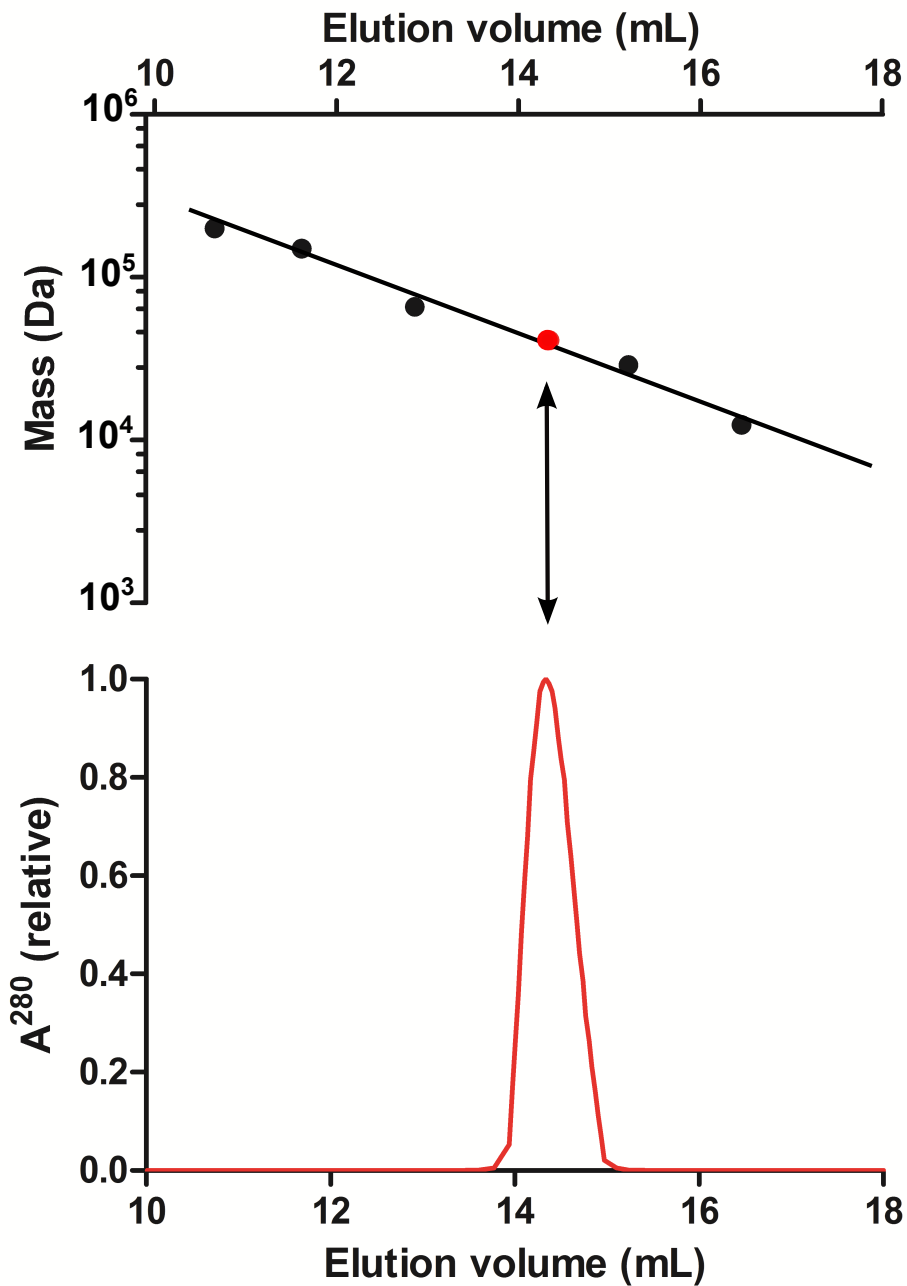

Supplement: S3 Fig — The bottom panel illustrates the optical absorption (280 nm) of the effluent. The top panel illustrates a semi-logarithmic plot of molecular mass vs. elution volume. Black circles correspond to the following protein standards (mass is given in kDa in parentheses): cytochrome c (12.4), carbonic anhydrase (29), BSA (66.4), alcohol dehydrogenase (150), and β-amylase (200). The red circle plot corresponds to the elution volume of the main peak against the sequence-deduced mass for a dimer (41 kDa) of AtCinA. (PDF) [file pone.0174759.s003.pdf]

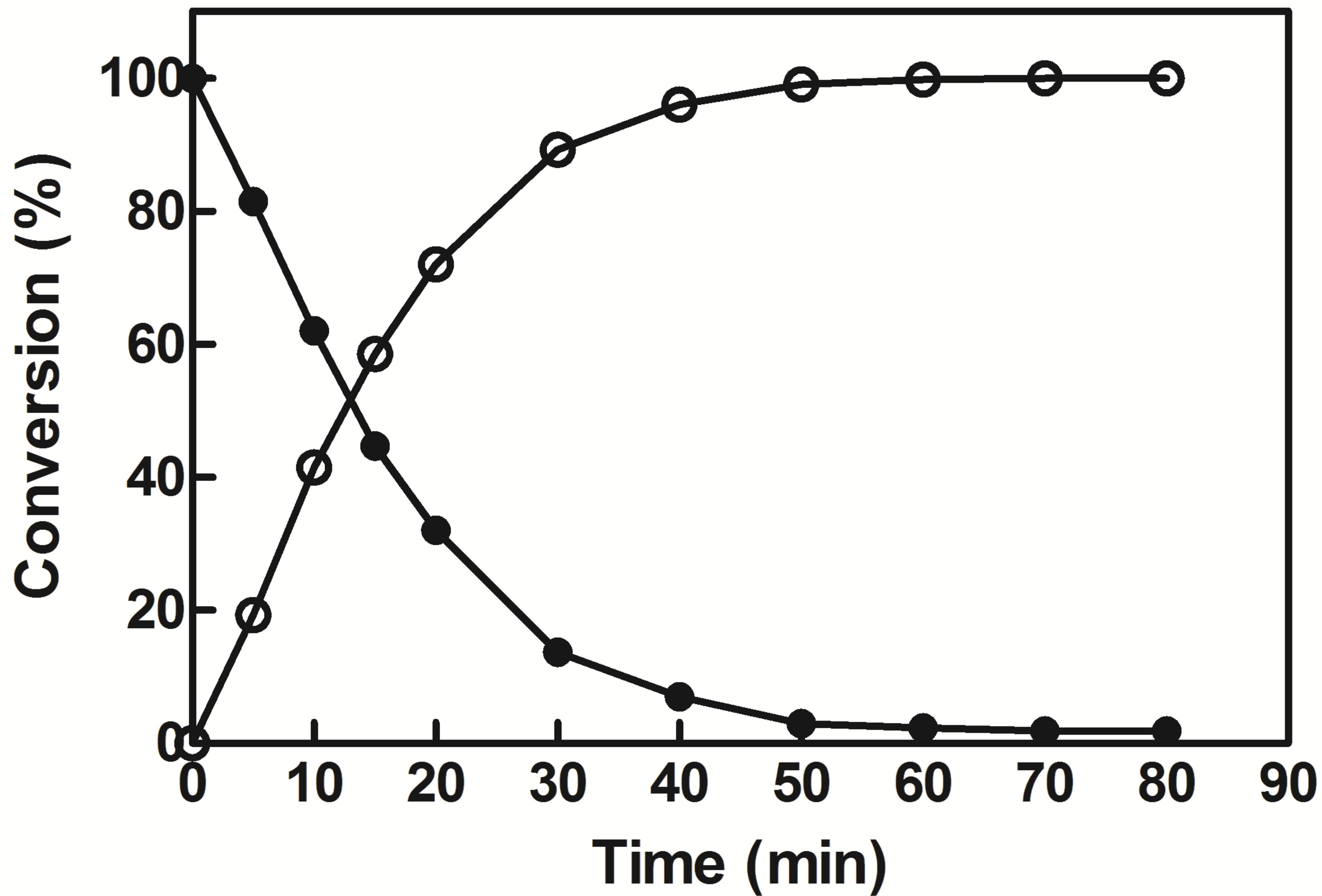

Supplement: S4 Fig — The reaction medium at 37 °C contained 10 mM of NMN and 140 nM AtCinA in 50 mM sodium phosphate buffer pH 7.5. (●) NMN and (○) NaMN. (PDF) [file pone.0174759.s004.pdf]

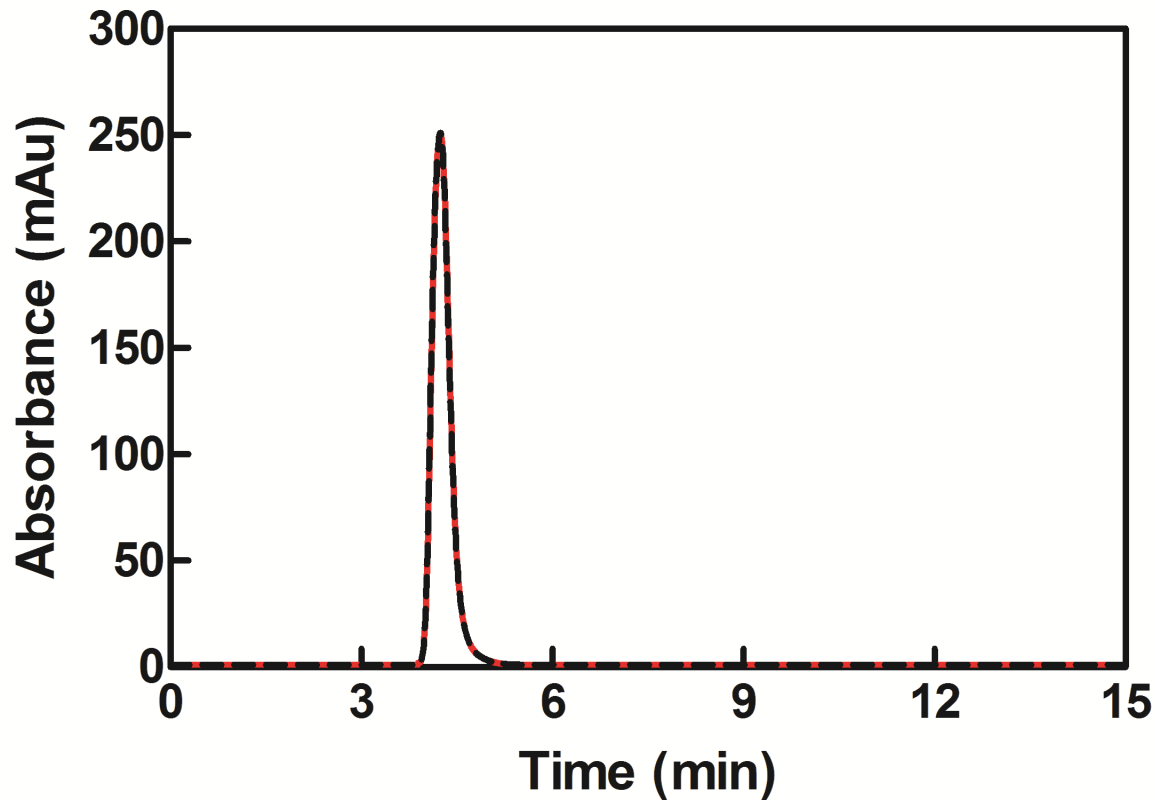

Supplement: S5 Fig — Dashed black line corresponds to NMN at zero time, and solid red line corresponds to NMN after 3 hours. No deamidation/decomposition was observed during this period. (PDF) [file pone.0174759.s005.pdf]

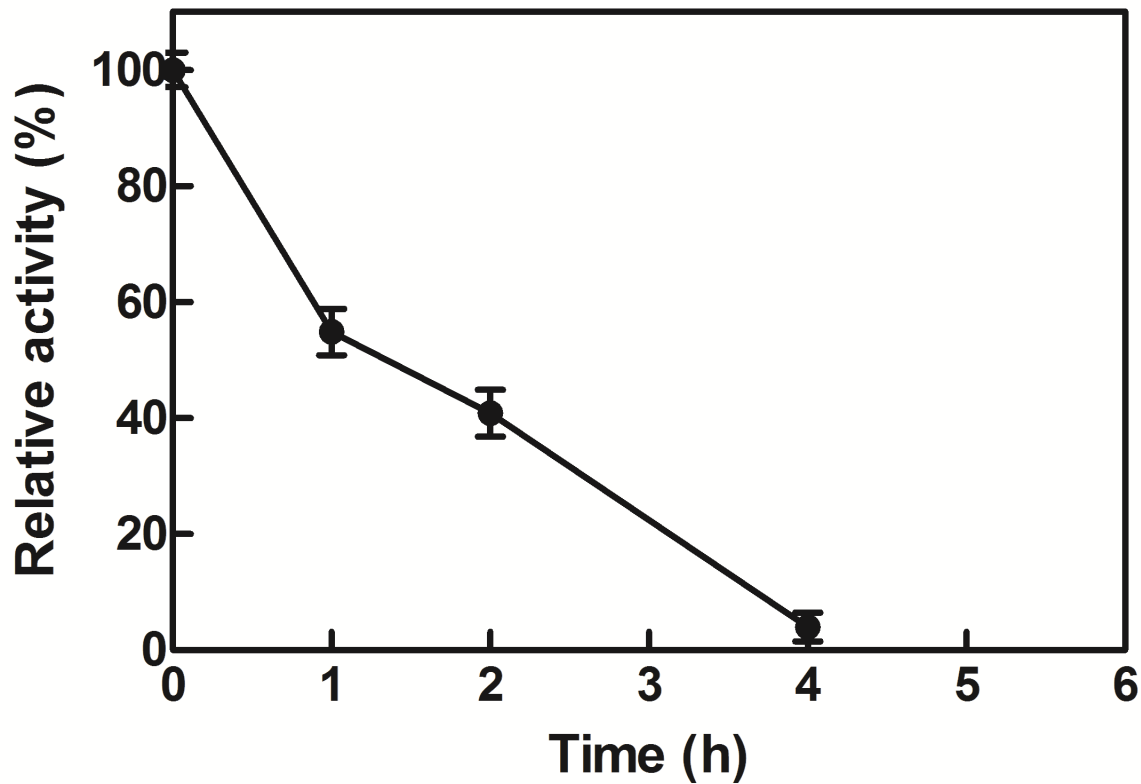

Supplement: S6 Fig — AtCinA was incubated for different periods of time at 80°C, and the activity was measured in the standard reaction conditions. (PDF) [file pone.0174759.s006.pdf]

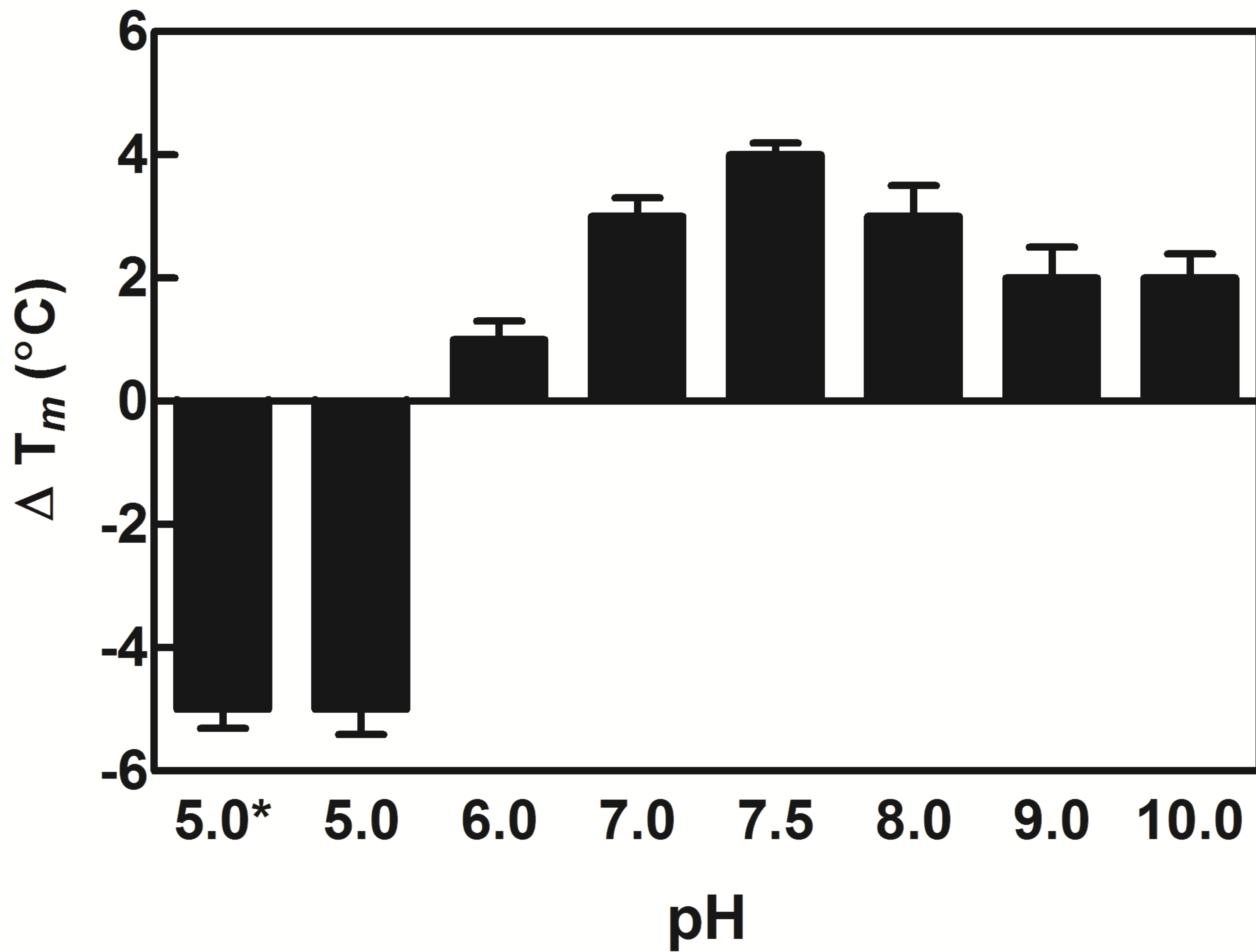

Supplement: S7 Fig — Differences in ΔTm were calculated by subtracting the MilliQ® water Tm value from the Tm values obtained for the enzyme at different pHs (50 mM sodium acetate pH 5.0, 50 mM sodium phosphate pHs 6.0–7.5, 50 mM Tris-HCl pH 8.0, and 50 mM glycine-NaOH pH 9.0–10.0). pH 5.0* indicates that the experiment was also carried out at 160 mM sodium acetate pH 5.0. (PDF) [file pone.0174759.s007.pdf]

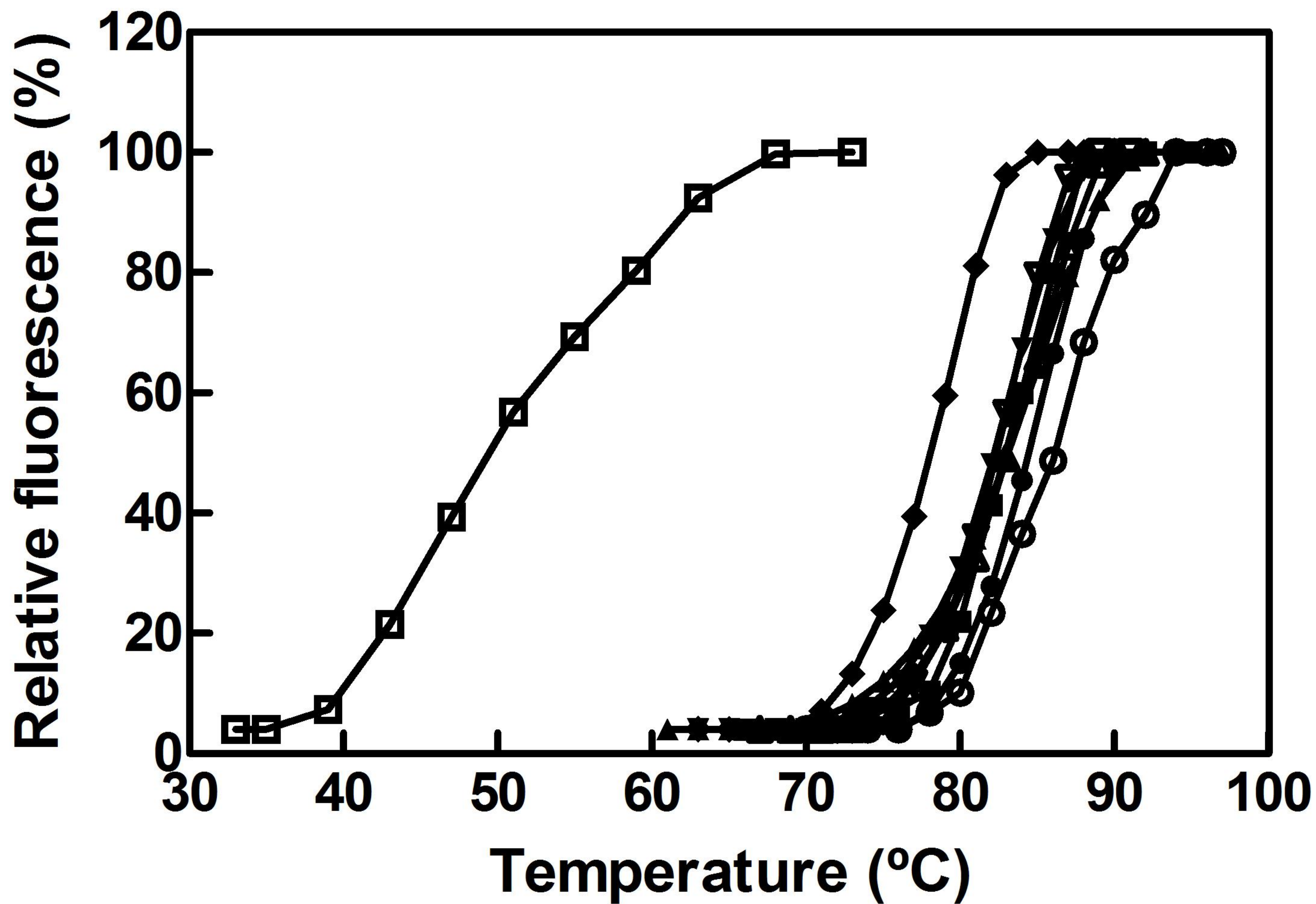

Supplement: S8 Fig — Melting temperature curves of purified enzyme (10 μg) were obtained in the presence of fluorescent probe SYPRO Orange. Wild type (●), S31A (○), C32A (△), S48A (■), Y58A (▲), Y58F (▼), K63A (◆), T105A (▽), and R145A (∕). (PDF) [file pone.0174759.s008.pdf]
