# Supplementary material for: Characterization and mutational analysis of a nicotinamide mononucleotide deamidase from Agrobacterium tumefaciens showing high thermal stability and catalytic efficiency
Source: PLoS One. 2017 Apr 7;12(4):e0174759. doi: 10.1371/journal.pone.0174759 (PMC5384747; doi:10.1371/journal.pone.0174759)
Supplement: S1 Table — (PDF) [file pone.0174759.s009.pdf]

Supporting Information

S1 Table. Sequences of primers used to clone AtCinA and its corresponding mutants.

	Forward	Reverse
**Wt**	5'-ccgcgctagcatgagccttttccccggagac-3'	5'-ctcgagctacaccgacccggcctgattgag-3'
**S31A**	5'-tctcgacggcggaagcctgcaccggagg-3'	5'-cctccggtgcaggcttccgccgtcgaga-3'
**C32A**	5'-cagtcctccggtggcgctttccgccgtc-3'	5'-gacggcggaaagcgccaccggaggactg-3'
**S48A**	5'-attgccggttccgccgccgtcgtcg-3'	5'-cgacgacggcggcggaaccggcaat-3'
**Y58A**	5'-gaccgtggttttgtcaccgctaccaatgacgccaagag-3'	5'-ctcttggcgtcattggtagcggtgacaaaaccacggtc-3'
**Y58F**	5'-ccgtggttttgtcacctttaccaatgacgccaaga-3'	5'-tcttggcgtcattggtaaaggtgacaaaaccacgg-3'
**K63A**	5'-cctataccaatgacgccgcgagagacatgctgggag-3'	5'-ctcccagcatgtctctcgcggcgtcattggtatagg-3'
**T105A**	5'-gccgtggcagtggccggcattgctg-3'	5'-cagcaatgccggccactgccacggc-3'
**R145A**	5'-gttacggagatatcggcgctactgaaatccggcttg-3'	5'-caagccggatttcagtagcgccgatatctccgtaac-3'
