# Supplementary material for: Characterization and mutational analysis of a nicotinamide mononucleotide deamidase from Agrobacterium tumefaciens showing high thermal stability and catalytic efficiency
Source: PLoS One. 2017 Apr 7;12(4):e0174759. doi: 10.1371/journal.pone.0174759 (PMC5384747; doi:10.1371/journal.pone.0174759)
Supplement: S2 Table — (PDF) [file pone.0174759.s010.pdf]

Supporting Information

S2 Table. Effects of metal ions and chemicals on AtCinA activity.

Compound	Relative activity (%)*	
	1 mM	10 mM
Mg^{2+}	98.3 ± 5.9	113.3 ± 3.0
Zn^{2+}	97.7 ± 1.4	71.7 ± 2.1
Ca^{2+}	99.9 ± 3.5	93.9 ± 4.1
Co^{2+}	92.9 ± 2.3	92.5 ± 5.9
Mn^{2+}	99.3 ± 1.9	115.8 ± 4.4
Li^{+}	94.7 ± 3.6	92.9 ± 1.4
EDTA	102.0 ± 4.0	95.2 ± 2.0
PMSF	74.3 ± 4.3	72.7 ± 0.9
Urea	109.1 ± 1.7	99.2 ± 3.0
2-mercaptoethanol	89.5 ± 5.2	92.5 ± 2.4
SDS	90.9 ± 2.3	0

*The activity of AtCinA without addition of any compound in the reaction mixture was defined as 100%.
